# Novelty in hypertension in children and adolescents: focus on hypertension during the first year of life, use and interpretation of ambulatory blood pressure monitoring, role of physical activity in prevention and treatment, simple carbohydrates and uric acid as risk factors

**DOI:** 10.1186/s13052-016-0277-0

**Published:** 2016-07-16

**Authors:** Mirella Strambi, Marco Giussani, Maria Amalia Ambruzzi, Paolo Brambilla, Ciro Corrado, Ugo Giordano, Claudio Maffeis, Silvio Maringhin, Maria Chiara Matteucci, Ettore Menghetti, Patrizia Salice, Federico Schena, Pietro Strisciuglio, Giuliana Valerio, Francesca Viazzi, Raffaele Virdis, Simonetta Genovesi

**Affiliations:** Gruppo di Studio Ipertensione Arteriosa Società Italiana di Pediatria, Rome, Italy; Dipartimento di Biologia Molecolare e dello Sviluppo, Università di Siena, Siena, Italy; ASL Milano 1, Novate Milanese Ollearo 2, 20155 Milan, Italy; ASL Milano 2, Melegnano, Italy; UOC Nefrologia Pediatrica A.R.N.A.S. Civico, Di Cristina e Benfratelli, Palermo, Italy; Alta Specializzazione Ipertensione Arteriosa, UOS Medicina dello Sport, Dipartimento Medico-Chirurgico di Cardiologia Pediatrica, Ospedale Pediatrico Bambino Gesù, Rome, Italy; UOC Pediatria ad Indirizzo Dietologico e Malattie del Metabolismo Azienda Ospedaliera Universitaria Integrata, Verona, Italy; Società Italiana Nefrologia Pediatrica, Milan, Italy; Ospedale Pediatrico Bambino Gesù IRCCS, Rome, Italy; Cardiologia Perinatale e Pediatrica, UOC Malattie Cardiovascolari, Fondazione IRCCS Ca’ Granda Ospedale Maggiore Policlinico, Milan, Italy; Società Italiana Cardiologia Pediatrica, Florence, Italy; Neonatologia, Fondazione IRCCS Ca’ Granda Ospedale Maggiore Policlinico, Milan, Italy; Dipartimento di Scienze Mediche Translazionali, Università Federico II Napoli, Naples, Italy; Dipartimento di Scienze Motorie e del Benessere, Università degli Studi di Napoli Parthenope, Naples, Italy; Dipartimento di Medicina Interna, Università di Genova e IRCCS AOU San Martino-IST, Genoa, Italy; Dipartimento Scienze Biomediche, Biotecnologiche e Traslazionali - S.Bi.Bi.T. Università di Parma, Parma, Italy; Dipartimento di Medicina e Chirurgia, Università di Milano Bicocca, Monza, Italy; Dipartimento di Scienze Cardiovascolari, Neurologiche e Metaboliche, Ospedale S. Luca, IRCCS, Istituto Auxologico Italiano, Milan, Italy; Società Italiana Ipertensione Arteriosa, Milan, Italy

## Abstract

The present article intends to provide an update of the article “Focus on prevention, diagnosis and treatment of hypertension in children and adolescents” published in 2013 (Spagnolo et al., Ital J Pediatr 39:20, 2013) in this journal. This revision is justified by the fact that during the last years there have been several new scientific contributions to the problem of hypertension in pediatric age and during adolescence. Nevertheless, for what regards some aspects of the previous article, the newly acquired information did not require substantial changes to what was already published, both from a cultural and from a clinical point of view. We felt, however, the necessity to rewrite and/or to extend other parts in the light of the most recent scientific publications. More specifically, we updated and extended the chapters on the diagnosis and management of hypertension in newborns and unweaned babies, on the use and interpretation of ambulatory blood pressure monitoring, and on the usefulness of and indications for physical activity. Furthermore, we added an entirely new section on the role that simple carbohydrates (fructose in particular) and uric acid may play in the pathogenesis of hypertension in pediatric age.

## Background

A few years ago we published in this journal an article on prevention, diagnosis and treatment of hypertension in children. During the last years there have been several new scientific contributions to the problem of hypertension in pediatric age and during adolescence. For this reason we felt the necessity to rewrite and/or to extend some parts of the previous article in the light of the most recent scientific publications.

### Hypertension in newborns and unweaned babies

**Fig. 1:**
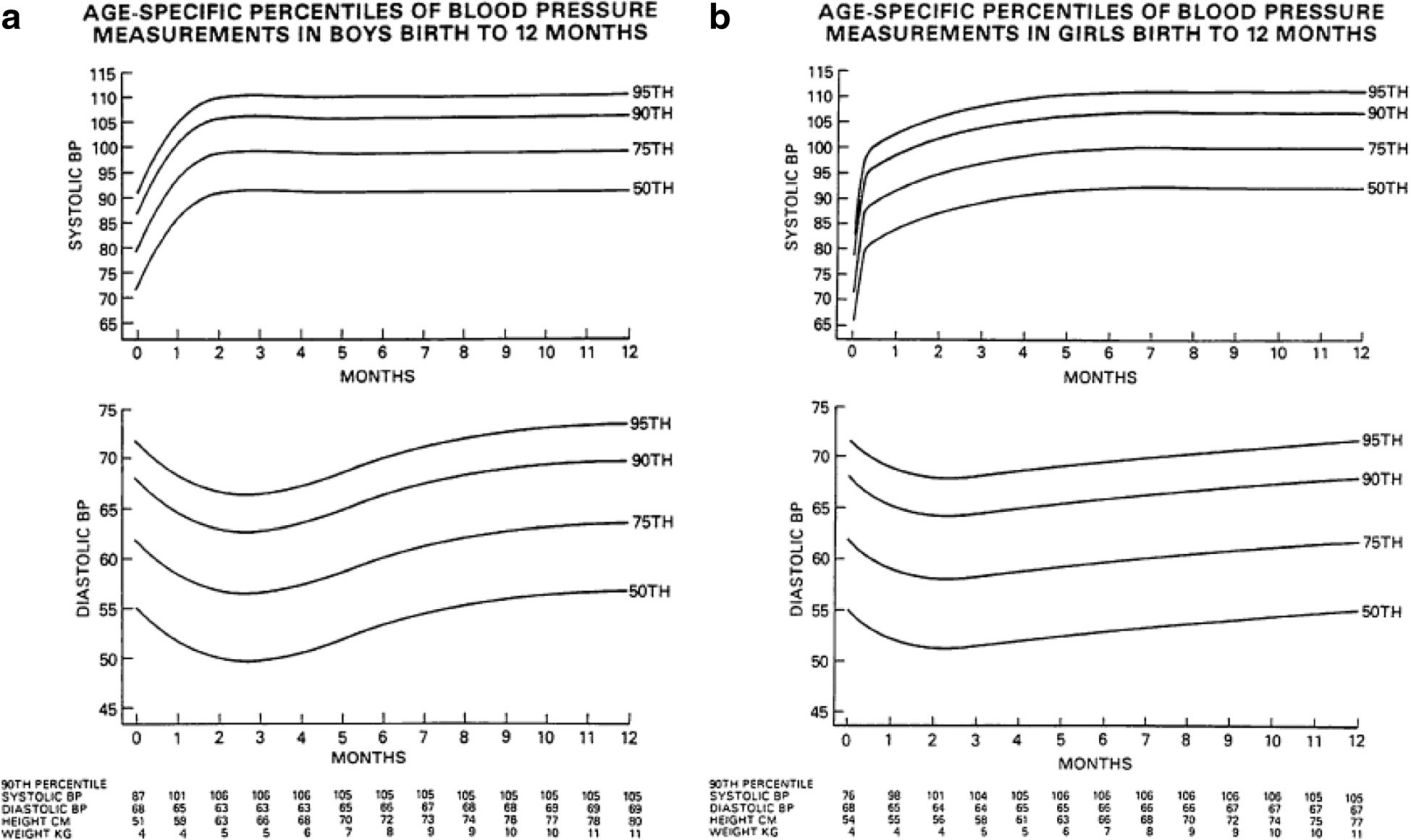
Percentiles of systolic and diastolic blood pressure from birth to 12 months of age Task Force on Blood Pressure Control in Children. Modified from Pediatrics 1987 [1]

The incidence of hypertension in healthy newborn infants is considered so low that blood pressure measurement is not recommended among the procedures to be performed during the first days of life; instead, the incidence appears to be higher (about 1-2 %) in newborns hospitalized in intensive care units. Reference values are based on surveys on a limited number of cases such as the American Task Force [1], or on a more numerous population in the study by Kent et al. published in 2007 in Pediatric Nephrology [2]. A recent review [3] of 43 studies confirms that sufficient data for the definition of normal blood pressure values in neonates are lacking, and that there are no follow-up data allowing the definition of those blood pressure values that, due to risk of organ damage, would require pharmacological treatment. Blood pressure values seem to be inversely proportional to gestational age and birth weight, increase in full-term newborns during of the first week of life, and increase during the first 15 days in early preterm infants (gestational age less than 31 weeks) [4, 5] and then seem to level off. These different behaviors are presumably due to the late closure of the ductus arteriosus and to the slower reduction of pulmonary resistances in preterm infants. In full-term babies blood pressure seems mainly associated with weight, whereas in preterm babies it appears to be related to gestational age. The measure of blood pressure that is generally used as a reference during the first month of life is mean blood pressure, as it best represents the overall hemodynamic situation at this stage. Mean blood pressure is obtained by summing the diastolic and 1/3 of the systolic blood pressure values. Among the available published surveys [6–10], both for preterm and for full-term babies we believe that currently it is advisable to use the study by Dionne that provides blood pressure values corresponding to the 50^th^, 95^th^, and 99^th^ percentile at 15 days of age according to gestational age [10] (Table 1). The definition of the 99^th^ percentile is important, because it distinguishes patients that need closer diagnostic examination and sometimes pharmacological therapy from those with borderline blood pressure values for whom an accurate follow-up may be sufficient. Regarding blood pressure progression with time in the infants, reference values for systolic and diastolic blood pressure during the first 12 months of life are reported in Fig. 1 [1].

**Table 1:** Gestational age and blood pressure values in newborns

GA (weeks)	50^th^ percentile			95^th^ percentile			99^th^ percentile		
	SBP	DBP	MBP	SBP	DBP	MBP	SBP	DBP	MBP
44	88	50	63	105	68	80	110	73	85
42	85	50	62	98	65	76	102	70	81
40	80	50	60	95	65	75	100	70	80
38	77	50	59	92	65	74	97	70	79
36	72	50	57	87	65	72	92	70	77
34	70	40	50	85	55	65	90	60	70
32	68	40	49	83	55	64	88	60	69
30	65	40	48	80	55	63	85	60	68
28	60	38	45	75	50	58	80	54	63
26	55	30	38	72	50	57	77	56	63

Modified from Dionne et al. [10]

*GA* gestational age, *SBP* systolic blood pressure, *DBP* diastolic blood pressure, *MBP* mean blood pressure

The method used for measuring blood pressure is an important factor. In newborns, when invasive recording of blood pressure is impossible, the oscillometric method is almost universally accepted as being reliable. The majority of measuring errors is caused by the use of inappropriate cuffs, a mistake that occurs in older children as well. Whereas in children it is advised that the width of the cuff be 40 % of the circumference of the arm, in newborn babies it has been shown that by using a cuff whose width equals 50 % of the arm circumference it is possible to detect blood pressure values that correspond with the intra-arterial ones [11]. It has also been demonstrated that the choice of the cuff after actual measurement of the arm’s circumference is much more accurate than the choice based on visual assessment, even if done by expert health workers [12]. Therefore the measurement of the arm circumference is an essential aspect of blood pressure assessment in neonates.

We can conclude that blood pressure measurement shouldn’t routinely be done in healthy newborns, but we also think that is mandatory to monitor blood pressure in neonatal intensive care unit, particularly in subjects submitted to mechanical ventilation, in sub-intensive care and whenever a newborn is at high risk for hypertension, due to either sickness or syndromic clues. The potential harms caused by false diagnosis of hypertension must be kept in mind and avoided by repeated measurements, performed by expert health workers.

#### Causes of hypertension in neonates

Neonatal hypertension is almost always secondary to an underlying disease or to a pre-existing clinical condition and is generally diagnosed in neonates hospitalized in the intensive care unit. Among the risk factors associated with hypertension, catheterization of the umbilical artery seems to have great importance and, as emerges from several studies [13–17] frequently causes aortic or renal thrombosis. Some prenatal factors are found to be related to an increased incidence of neonatal hypertension, and most of all maternal hypertension (gestational or prior to pregnancy) and the use of corticosteroids by pregnant women [18] seem to be implicated. The main causes of neonatal hypertension can be classified in the following groups: renal parenchymal, renovascular, due to compression of renal vessels or aorta (abdominal masses, defects of closure of the abdominal wall), cardiac, iatrogenic, malformations, endocrine, neurologic, respiratory (Table 2). The etiology of hypertension is quite different for preterm compared to full-term babies: in preterms the clearly most prevalent causes are bronchodysplasia and iatrogenic factors. Bronchodysplasia has been identified as a cause of systemic hypertension by Abman in 1984 and this observation has been confirmed by later studies [19–21]. The incidence of hypertension among neonates with very low birth weight and bronchodysplasia is twice compared to newborns without bronchodysplasia and appears to be associated with the severity of the pulmonary disease [21]. The pathogenetic mechanisms that connect bronchodysplasia and hypertension are not quite clear. Possible mechanisms that are especially taken into consideration are impaired neurohumoral regulation, increased levels of catecholamines, angiotensin and antidiuretic hormone, but also merely the frequent use of umbilical arterial cathetherization in these patients [22]. Many drugs that are commonly administered to preterm infants can induce hypertension; dexamethasone and other corticosteroids used to treat chronic pulmonary diseases, catecholamines (dopamine, adrenaline), xanthines (caffeine and theophylline), curare-mimetics (pancuronium) and mydriatic eye drops (phenylephrine). The hypertension induced by these drugs may in some cases last even for a certain time after the interruption of the drug itself. Several studies have shown an higher incidence of systemic hypertension in preterm infants treated for patent ductus arteriosus; in these cases the elevated blood pressure may be related to the possible nephrotoxic effect of indomethacin, to renal hypoperfusion due to diastolic theft caused by the patent ductus, to increased stroke volume following closure of the ductus or, finally, to thromboembolic events [18, 20, 23] In full-term infants hypertension is most frequently associated with renal diseases; other causes are observed with more or less equivalent prevalence. Newborns suffering from some malformation syndromes (DiGeorge, CHARGE, VACTERL, Congenital Rubella, Potter) may sometimes present hypertension as a consequence of renal, cardiac or vascular malformations.

**Table 2:** Causes of neonatal hypertension

Renovascular
Renal artery stenosis
Renal artery thrombosis
Renal venous thrombosis
Renal parenchymal disease/obstructive uropathy
Polycystic kidney disease
Acute tubular necrosis
Nephrocalcinosis
Severe vesicoureteral reflux
Stenosis of ureteropelvic junction
Posterior urethral valve
Ureterocele
Other causes of acute and chronic renal insufficiency
Cardiac
Aortic coarctation
Aortic arch reconstruction
Patent ductus arteriosus
Endocrine
Congenital adrenal hyperplasia (17 alpha or 11 beta hydroxylase deficiency)
Hyperthyroidism
Hyperaldosteronism
Hypercalcemia
Neurologic
Intracranial hypertension
Seizures
Intraventricular hemorrhage
Abstinence syndrome from opioid withdrawal
Pain
Neoplasia
Neuroblastoma
Wilms tumor
Pheochromocytoma
Closure of abdominal wall defect
Gastroschisis
Giant omphalocele
Iatrogenic
Dexamethasone and other corticosteroids
Methylxanthines (caffeine, theophylline)
Vasoactive amines (dopamine, adrenaline)
Bronchodilators
Phenylephrine
Parenteral nutrition (volume overload, sodium, calcium)
Extracorporeal respiratory assistance
Malformation syndromes
DiGeorge
Potter
Congenital Rubella
CHARGE
VACTERL

#### Clinical approach

Whenever an elevated blood pressure is detected, the method of measurement should be verified and it would be necessary, at least once, to measure the blood pressure at all four extremities in order to exclude the presence of aortic coarctation. Subsequently the most frequent causes of hypertension (Table 3) are to be excluded and it should be evaluated if the currently installed therapy involves potentially hypertensive drugs. In infants the malformation syndromes are often characterized by the vague nature of their signs and therefore it is important to pay close attention to facies, nasal bridge, positioning of the ears, the possible presence of a nuchal fold and to ask for genetic counselling in case of doubt. Careful examinations of the heart and abdomen are crucial. Serum levels of creatinine and electrolytes should be determined and a urine test should be performed in order to exclude renal parenchymal disorders. Renal vein thrombosis should be suspected in neonates presenting haematuria, thrombocytopenia, palpable renal mass or anuria. Ultrasonography is usually sufficient for the diagnosis: typical findings include an enlarged kidney, increased echogenicity around interlobular vessels and loss of cortico-medullary differentiation. The direct visualization of the thrombus in the renal vein or in the inferior vena cava is often possible. In case of renal vein thrombosis, the doppler ultrasound scan demonstrates an absent flow in the renal vein.

**Table 3:** Guiding criteria for the diagnosis of neonatal hypertension

The most frequent prenatal causes of neonatal hypertension are:
1. repeated use of steroids by mother
2. maternal diabetes mellitus
3. maternal hypertension
The most frequent postnatal causes of neonatal hypertensione are:
1. thromboembolism associated with the placement of an umbilical cathether, involving an acute risk presumably due to endothelial damage and a chronic risk related to the time the cathether stays in place
2. renal parenchymal disease, both congenital (eg. polycystic kidney disease) and acquired
3. bronchopulmonary dysplasia (CLD)

The presence of thrombophilia may confirm the hypothesis of renal vein thrombosis. Only as a second step and if the clinical history suggests to do so, cortisol and aldosterone levels and the thyroid function should be assessed. Plasma renin activity is normally very high in newborns, and consequently it is not to be assessed, unless a marked hypokalemia is found that may possibly be related to a genetic anomaly in the tubular sodium management. Echo-Doppler examination of the renal vessels is essential for excluding renal malformations and aortic or renal intravascular thrombi. Until a few years ago, angiography was considered the gold standard for the diagnosis of renal artery stenosis due to fibrodysplasia, but was difficult to carry out in newborns. The search for less invasive diagnostic methods has recently resulted in obtaining a very high diagnostic sensitivity and specificity by combining color-Doppler technique to nuclear magnetic resonance or to a computed tomography scan with contrast. This latter method provides better definition, even though at a high level of toxicity [24, 25].

#### Treatment

A wide variety of drugs is being used in the treatment of hypertension in neonates, but the indications provided by literature are exclusively based on isolated surveys or on expert’s opinions and no controlled studies on efficacy an safety are available. For these reasons the use of antihypertensive agents in this age range is generally not approved by the regulatory authorities (AIFA, EMEA, FDA) and should be considered off-label. There is not even a unanimous consensus on which blood pressure values would benefit from treatment, as it is not well known what are the harmful effects of persistently high blood pressure levels at this age. In any case it is appropriate to consider pharmacological treatment when blood pressure repeatedly exceed the 99^th^ percentile. Before starting therapy the possibility to remove the cause of hypertension should be examined (iatrogenic causes or conditions that can be treated surgically, such as coarctation of the aorta, some neoplasias or certain urologic diseases). Instead, when it is necessary to resort to medical treatment it is advisable to use intravenous administration in all the emergency situations in which it is beneficial to allow quick dose changes according to the blood pressure response; oral administration is indicated in less urgent cases or as long-term treatment after having succeeded in controlling the hypertension by intravenous therapy. Among the different classes of antihypertensive drugs, ACE-inhibitors are the most frequently used in newborns, especially when renal diseases are present [26]. However, some authors [27] have recommended not to use ACE-inhibitors in preterm neonates because of their possible negative effects of the development of the kidney. Moreover, in these particular children the reaction to these drugs is rather unpredictable and there is a certain risk of causing profound hypotension with possible renal failure and severe neurological consequences. In particular, in the case of captopril in preterm neonates it is advised to use initial doses that are much lower than those used in full-term babies [28]. Diuretics (hydrochlorothiazide, furosemide, spironolactone) are also widely used in newborns and should be the first choice treatment in case of bronchodysplasia, as they show positive effects on pulmonary mechanics. In this kind of patients, and generally in all preterm neonates with cardiorespiratory instability, beta-blockers should be used with much caution, due to the risk of episodes of severe apnea and bradycardia [29]. Arteriolar vasodilators (especially hydralazine) and calcium antagonists are found to be most frequently used in some America case records, both in acute situations and as long-term treatment [20, 30]. Table 4 summarizes the oral and intravenous drugs that can be used, indicating doses, frequency and mode of administration, and information on which precautions to take and on possible side effects.

**Table 4:** Drugs useful in the treatment of neonatal hypertension

Drug	Class	Dose	Route	Comments
Intravenous agents^a^:				
Diazoxide	Vasodilator (arteriolar)	2–5 mg/kg/dose	Rapid bolus injection	Slow injection is ineffective. Duration unpredictable. May cause rapid hypotension.
Enalaprilat	ACE-inhibitor	15 ± 5 μg/kg/dose; repeat after 8–24 h	Injection over 5–10 min	May cause prolonged hypotension and acute renal insufficiency.
Esmolol	β-blocker	100–300 μg/kg/min	IV infusion	Very short-acting. Constant infusion necessary.
Hydralazine	Vasodilator (arteriolar)	Bolus: 0.15–0.6 mg/kg/dose every 4 h. Drip: 0.75–5.0 μg/kg/min	IV bolus or infusion	Frequently causes tachycardia.
Labetalol	α- and β-blocker	0.20–1.0 mg/kg/dose 0.25–3.0 mg/kg/h	IV bolus or constant infusion	Heart failure and BPD are relative contraindications.
Nicardipine	Calcium antagonist	1–3 μg/kg/min	Constant infusion	May cause reflex tachycardia.
Sodium nitroprusside	Vasodilator (arteriolar and venous)	0.5–10 μg/kg/min	Constant infusion	In case of prolonged therapy (>72 h) or renal failure thiocyanate toxicity may occur.
Drug	Class	Dose	Interval	Comments
Oral agents^a^				
Captopril	ACE-inhibitor	0.01–0.5 mg/kg	TID	Monitor serum creatinine and potassium.
Clonidine	Central α-agonist	0.05–0.1 mg/kg?	BID-TID	Side effects: sedation, dryness of the mucosa. Abrupt interruption may cause rebound hypertension.
Hydralazine	Vasodilator (arteriolar)	0.25–1.0 mg/kg (max 7.5 mg/kg per day)	TID-QID	Frequent side effects: fluid retention and tachycardia. Lupus-like syndrome may occur in slow acetylators.
Isradipine	Calcium antagonist	0.05–0.15 mg/kg	QID	Useful for both chronic and acute hypertension.
Amlodipine	Calcium antagonist	0.1-0.3 mg/kg	BID	Causes sudden hypertension less frequently than isradipine.
Nifedipine	Calcium antagonist	0,25–0,5 mg/kg Max. dose: 1–2 mg/kg per day	Repeat every 4–6 h	Hypotensive response poorly predictable.
Minoxidil	Vasodilator (arteriolar)	0.1–0.2 mg/kg	BID-TID	The most potent oral vasodilator. Hypertrichosis in case of prolonged use.
Propranolol	β-blocker	0.5–1.0 mg/kg	TID	Maximum dose to be defined according to heart rate (up to 8–10 mg/kg if no bradycardia). Not to be used in infants with BPD.
Atenolol	β-blocker	0.8–1 mg/kg Max. dose 2 mg/kg per day	QD	
Labetalol	α- and β-blocker	1.0 mg/kg	BID-TID	Monitor heart rate. Not to be used in infants with BPD.
Spironolactone	Aldosterone antagonist	0.5–1.5 mg/kg	BID	Potassium-sparing. Check serum electrolytes. May take several days to reach maximum effectiveness.
Hydrochlorothiazide	Thiazide diuretic	1–3 mg/kg	QID	Check serum electrolytes.
Chlorothiazide	Thiazide diuretic	5–15 mg/kg	BID	Check serum electrolytes.

^a^modified from Flynn, [6]

#### Organ damage

Hypertension is the main cause of myocardial hypertrophy with consequent fibrosis and dilatation; in adults such conditions are associated with cardiac and non-cardiac morbidity and untimely mortality [31–33] Antihypertensive therapy causes a reduction in blood pressure and cardiac mass. However, it has never been demonstrated that normalization of blood pressure and cardiac mass brings cardiovascular risk to the same level of subjects who were never hypertensive; what is more, it seems that hypertensive patients, even though treated correctly and achieving efficient reduction of blood pressure levels, still remain at increased risk compared to normotensives. The few available studies on this subject in pediatric age seem to support this theory [34, 35]. Thus it is important, even in young children, to prevent hypertension or at least prevent left ventricular hypertrophy. Cardiac hypertrophy can be easily assessed by echocardiography that, by measuring the wall thicknesses and dimension of the chambers, allows to determine cardiac mass with an appropriate formula which provides a good correspondence with necropsy left ventricular mass [36]. Cardiac mass as expressed in grams is indexed according to weight and/or height of the subject. Scientific knowledge on hypertension-induced organ damage in childhood is scarce, but it is known that also children react to increases in blood pressure with increases in cardiac mass, and thus presumably suffering all the same consequences and risks as those described for adults [37]. Information on neonates is even more scanty. The heart of newborn infants still has the capacity to increase the number of myocytes (hyperplasia). Within one year of age this capacity gradually diminishes. As the rate of growth of cardiac mass is inversely proportional to age, in a short time lapse severe hypertension can induce a considerable hypertrophy with a consequent rise in the associated risk of death in newborns and unweaned babies [38]. Even if shown in a limited number of subjects, an increase in mass of more than 100 % compared to baseline values may be associated with an increased mortality [39]. In conclusion, a rapid development of ventricular hypertrophy represents a negative prognostic factor of survival and requires a timely and efficient treatment, especially when considering that blood pressure reduction in this age range seems to bring about an correspondingly rapid regression of the hypertrophy. The risks associated to increased cardiac mass and the age-related high rate of hypertrophy development, make careful cardiologic follow-up of cases of severe hypertension in infants and unweaned babies quite crucial. Arterial stiffness evaluated by pulse wave velocity and ultrasonography measurement of intimal media thickness are important independent predictors of cardiovascular events in adults and indicators of cardiovascular risk in childhood [40, 41]. Both pulse wave velocity and intimal media thickness have been shown to be reliably ascertained in newborns with good reproducibility, but normal reference values are still lacking. So it is difficult to distinguish between the normal progressive structural changes in the arterial wall in this age group and differences due to the systemic blood pressure [42, 43]. Furthermore recent data show significantly increased intimal media thickness and pulse wave velocity values during infancy in growth restricted foetuses, not always related to a significant trend because of high blood pressure values, supporting that possibility of fetal cardiovascular programming leading later in life on to hypertension [44].

#### Prevention of primary hypertension: programming

In recent years it has been established that primary hypertension, besides being controlled by an important genetic component, is influenced by environmental stimuli according to the concept of programming; i.e. the process by which a stimulus or a lesion to an organism during a critical period of its development determines long-lasting or even permanent effects. While the duration of the stimulus is certainly important, the period in life in which it occurs is even more critical. If common stimuli affecting blood pressure such as stress, diet, drugs, are present during intrauterine life or during the first year of life, they may alter the programming and determine permanent changes in blood pressure [45]. There is some debate about the pathogenetic mechanism; the initial hypothesis that still stands is the so-called unifying pathway by Woods [46], according to which environmental and placental factors, such as maternal protein restriction, alterations in the placental or uterine circulation, or placental insufficiency, which all determine low birth weight, may cause hypertension in adulthood due to activation of the renin-angiotensin system as a consequence of a low number of nephrons. Furthermore, according to the hypothesis developed by Barker [47], scarce intrauterine growth would cause a deterioration in insulin resistance, and in turn, elevated levels of insulin have been associated with increased blood pressure also in children [48]. Several studies report that adolescents and adults who were born prematurely have a mean blood pressure that is higher than the one observed in full-term peers or show an increased pressure load (number of ambulatory blood pressure measurements beyond normal values), both indicative of a predisposition for the development of hypertension [49–51]. More recent data suggest that even children of premature parents, though they themselves were born after a full-term delivery, present modest abnormalities in their cardiovascular disposition (higher heart rate or elevated blood pressure values, yet remaining within the normal range). These observations seem to confirm that changes induced by environmental stimuli can be inherited by the following generations [52]. Another important factor is represented by the type of nutrition during the first year of life. One of the beneficial effects of breastfeeding is the reduction of blood pressure at later age, but also mixed feeding seems to be effective. In fact, in 2001 Singhal et al. reported in Lancet [53] that diastolic blood pressure at 14–16 years of age is inversely proportional to the percentage of mother’s milk in the diet during the first months of life; the more mother’s milk had been consumed, the lower was diastolic blood pressure. It is known that potassium inhibits the renin-angiotensin system. The high content of potassium in vegetables, but also potassium in formula milk, the quantity of which may vary a lot according to the type of milk, may affect blood pressure values [54, 55]. All these studies emphasize the importance of very early preventive strategies, starting as early as in pregnancy, involving lifestyle and diet interventions in order to maintain a healthy condition in the long term.

### Ambulatory blood pressure monitoring in pediatric hypertension: how and when to use it

In 2008 the first recommendations were published for the use and interpretation of 24-h ambulatory blood pressure monitoring (ABPM) in children and adolescents [56]. As specified in the introduction of that publication, these “recommendations” were meant to be considered experts’ opinion, because the limited number of clinical data did not allow to define them as “guidelines”. The document, though emphasizing the importance of ABPM in the assessment of blood pressure values in children and adolescents, pointed out some critical points concerning its use. In particular, the lack of solid reference values for normal subjects was underlined; the tables reported in the recommendations had been derived from the only study that had provided sufficient data for the creation of specific nomograms, subdivided according to gender and age, or gender and height [57]. The study population (949 subjects) was composed of children of a single ethnicity (Caucasian) within an age range of 5–16 years. The authors confirmed the fact that diagnosis of hypertension in pediatric age should be based on repeated blood pressure measurements performed in the doctor’s office as indicated by the Fourth Report on the Diagnosis, Evaluation and Treatment of High Blood Pressure in Children and Adolescents [58], but they also suggested useful criteria for the classification of blood pressure values obtained by ABPM. Besides referring to the percentiles proposed by Wühl, the recommendations added the concept of blood pressure load, to be intended as the percentage of measurements revealing pressure levels above the 95^th^ percentile [59]. A blood pressure load exceeding 25 % is considered elevated. When the measured percentage of pathological blood pressure values is above 50 % this outcome is an indication of severe hypertension (at risk of organ damage) [60]. In short, the document advised the use of ABPM for the confirmation of hypertension (identification of white coat hypertension or masked hypertension), for the assessment of blood pressure variability (presence of physiological nocturnal fall, dipping) and of blood pressure load, and to verify the efficacy of therapy in treated subjects, especially in secondary forms of hypertension. Six years later an update of these recommendations was published [61]. In this update the authors implement the previous recommendations with new data emerging from the recent literature and propose a revision of the classification scheme that was published in the 2008 document, more accurately defining children with prehypertension (Table 5); furthermore they introduce the concept of diastolic hypertension. In the introduction it is again declared that this document should be considered an experts’ opinion, even though supported by more important clinical evidence compared to the preceding version. The blood pressure nomograms advised for the interpretation of ABPM values remain identical compared to the 2008 recommendations, as actually no new studies on larger and differentiated populations to define new reference values had been performed in the meantime. Tables 6, 7, 8 and 9 summarize ambulatory systolic and diastolic blood pressure reference values (mean values, daytime and nighttime, 90^th^ and 95^th^ percentiles) according to gender and height and to gender and age. The authors emphasize the necessity to more precisely identify the relationship between different ABPM patterns and the presence of organ damage. The new document more clearly explains a number of crucial points for the correct performance of ABPM in children; Our aim is to summarize and comment these points.

**Table 5:** Scheme for the classification of systolic and diastolic blood pressure values obtained by 24-h blood pressure monitoring in children

Classification	Office SBP/DBP ^a^	24-h ambulatory SBP/DBP ^b^	SBP or DBP load (%) ^b^
Normal blood pressure	<90^th^ percentile	<95^th^ percentile	<25
White coat hypertension	≥95^th^ percentile	<95^th^ percentile	<25
Prehypertension	≥90^th^ percentile	<95^th^ percentile	≥25
Masked hypertension	<95^th^ percentile	>95^th^ percentile	≥25
Hypertension	>95^th^ percentile	>95^th^ percentile	25–50
Severe hypertension	>95^th^ percentile	>95^th^ percentile	>50
Dipping subjects: mean nocturnal values at least 10 % lower than diurnal values			

^a^ The Fourth Report on the Diagnosis, Evaluation, and Treatment of High Blood Pressure in Children and Adolescents, [58]

^b^ Modified from Flynn JT et al. [61]

**Table 6:** Normal values for ambulatory BP (mmHg) for girls by height

Height (cm)	24 h		Day		Night	
	90^th^ p	95^th^ p	90^th^ p	95^th^ p	90^th^ p	95^th^ p
120	112/71	114/72	118/80	120/82	103/63	106/65
125	113/71	116/73	119/80	121/82	104/63	107/66
130	114/72	117/72	120/80	122/82	106/63	108/66
135	115/72	118/74	120/80	123/82	107/63	109/66
140	116/73	119/75	121/80	124/82	108/63	110/66
145	117/73	120/75	122/80	125/82	109/63	112/66
150	119/74	121/76	124/80	127/82	110/63	113/66
155	120/74	123/76	125/80	128/82	111/63	114/66
160	121/74	123/76	126/80	129/82	111/63	114/66
165	122/74	124/76	127/80	130/82	112/63	114/66
170	123/74	125/76	128/80	131/82	112/67	115/71
175	124/75	126/76	129/81	131/82	113/63	115/66

**Table 7:** Normal values for ambulatory BP (mmHg) for boys by height

Height (cm)	24 h		Day		Night	
	90^th^ p	95^th^ p	90^th^ p		90^th^ p	95^th^ p
120	114/74	117/77	122/80	125/82	103/61	106/63
125	115/74	118/77	122/80	125/82	105/61	108/61
130	116/74	119/77	122/80	122/82	106/62	110/64
135	117/74	120/77	123/80	126/82	108/63	111/65
140	118/75	121/77	123/80	126/82	109/63	113/65
145	120/75	123/77	124/79	127/81	111/64	114/66
150	121/75	124/77	125/79	128/81	112/64	116/66
155	123/75	126/77	127/79	130/81	113/64	117/66
160	124/75	127/77	129/79	133/81	114/64	118/66
165	126/75	129/78	132/80	135/82	116/64	119/66
170	128/75	131/78	134/80	138/82	117/64	121/66
175	130/75	133/78	136/80	140/83	119/64	122/66
180	131/76	134/78	138/81	142/83	120/64	124/66
185	133/76	136/78	140/81	144/84	122/64	125/66

**Table 8:** Normal values for ambulatory BP (mmHg) for girls by age

Age (years)	24 h		Day		Night	
	90^th^ p	95^th^ p	90^th^ p		90^th^ p	95^th^ p
5	112/72	115/74	118/80	121/82	105/66	109/69
6	114/72	116/74	120/80	122/82	106/65	110/68
7	115/72	118/74	121/80	123/82	107/65	111/67
8	116/72	119/74	122/80	124/82	108/64	112/67
9	117/73	120/74	122/80	125/82	109/64	112/67
10	118/73	121/75	123/79	126/81	110/64	113/66
11	119/73	122/75	124/79	127/81	110/63	114/66
12	120/74	123/76	125/80	128/82	110/63	114/66
13	121/74	124/76	126/80	129/82	111/63	114/66
14	122/74	125/76	127/80	130/82	111/63	114/65
15	123/75	125/77	128/80	130/82	111/63	114/65
16	123/75	126/77	129/82	131/82	111/63	114/65

**Table 9:** Normal values for ambulatory BP (mmHg) for boys by age

Age (years)	24 h		Day		Night	
	90^th^ p	95^th^ p	90^th^ p		90^th^ p	95^th^ p
5	114/72	116/74	120/79	123/81	103/62	106/65
6	115/73	118/75	121/79	124/81	105/63	108/66
7	116/73	119/75	122/80	125/82	106/64	110/67
8	117/73	120/75	122/80	125/82	108/64	111/67
9	118/73	121/75	123/80	126/82	109/64	112/67
10	119/73	123/75	124/80	127/82	110/64	113/67
11	121/74	125/76	126/80	129/82	111/64	115/67
12	124/74	127/76	128/80	132/82	113/64	116/67
13	126/74	130/76	131/80	135/82	115/64	119/67
14	129/75	133/77	134/80	138/82	118/64	121/67
15	132/75	136/77	137/81	141/83	120/64	123/66
16	135/76	138/78	140/81	144/84	123/64	126/66

ABPM: 24 h ambulatory blood pressure monitoring

Modified from Flynn JT et al. [61]

#### ABPM and organ damage

Several studies reported a correlation between blood pressure values observed in pediatric age and the presence of organ damage in adulthood, and in particular the presence of left ventricular hypertrophy, carotid intima-media thickening and increase in arterial stiffness [62–64]. ABPM appears to be more effective compared to office blood pressure measurements in identifying children at increased risk of developing organ damage. A study by Sorof [65] clearly shows diagnosis of hypertension in children on the basis of ABPM data is more significantly related to the prevalence of left ventricular hypertrophy than hypertension diagnosed by office blood pressure measurements. In a review of the National High Blood Pressure Education Program Working Group the risk of developing left ventricular hypertrophy, in terms of odds ratio, was 7.2 in hypertension diagnosed by ABPM vs 4.1 in hypertension based on office measurements [66]. In another study, the blood pressure values obtained by ABPM were shown to be superior in predicting LVH than both office and home (self-measurement) blood pressure values [67]. Furthermore, in several studies blood pressure values recorded by ABPM were associated with another well-known cardiovascular risk factor, namely carotid intima-media thickness [68, 69]. Litwin et al. [70] documented, in 72 children with essential hypertension, a correlation between intima-media thickness in the carotid and superficial femoral arteries and 24-h systolic blood pressure values. Finally, others authors documented a correlation between ABPM values and some parameters of artery distensibility and stiffness or with indices of endothelial dysfunction as flow-mediated or nitroglycerin-mediated dilation [71, 72].

#### Indications for the use of ABPM

Data provided by ABPM consist of the mean of systolic and diastolic blood pressure values recorded during 24 h and, separately, during waking hours (daytime mean) and during sleep (nighttime mean). The recording of nighttime blood pressure allows the determination of the presence of the physiological fall in blood pressure during sleep (dipping), which should be at least 10 % of the diurnal blood pressure mean. The absence of a nighttime fall in blood pressure and the presence of isolated nocturnal hypertension have been shown to be important prognostic factors in several forms of secondary hypertension, especially those due to renal damage [73–76]. Another important concept to use in the interpretation of a 24-h recording is pressure load. A correct definition of all these parameters requires an adequate identification of the actual periods of sleep and wakefulness in the patient.

#### ABPM and clinical condition

##### White coat hypertension

The American recommendations suggest to perform ABPM in children with elevated office blood pressure values in whom an excess reactivity to the measurements (white coat hypertension) is suspected, in order to confirm such possible diagnosis. However, as initial organ damage may already be present in subjects with white coat hypertension [77–80] in the last recommendations it is suggested to consider white coat hypertensives only those subjects with borderline or moderately elevated office blood pressure values, because subjects with even higher blood pressure could be real hypertensives, as demonstrated by Sorof [81]. An increase in left ventricular mass has in fact been demonstrated in children with white coat hypertension [77] and it has been shown that also subjects who occasionally have blood pressure values above the 95^th^ percentile (transitory hypertensives) may be at increased risk of presenting left ventricular hypertrophy [34]. For these reasons we believe that when normal ABPM values are observed in the presence of elevated office blood pressure values, these children should not be excluded from careful follow-up.

##### Masked hypertension

**Fig. 2:**
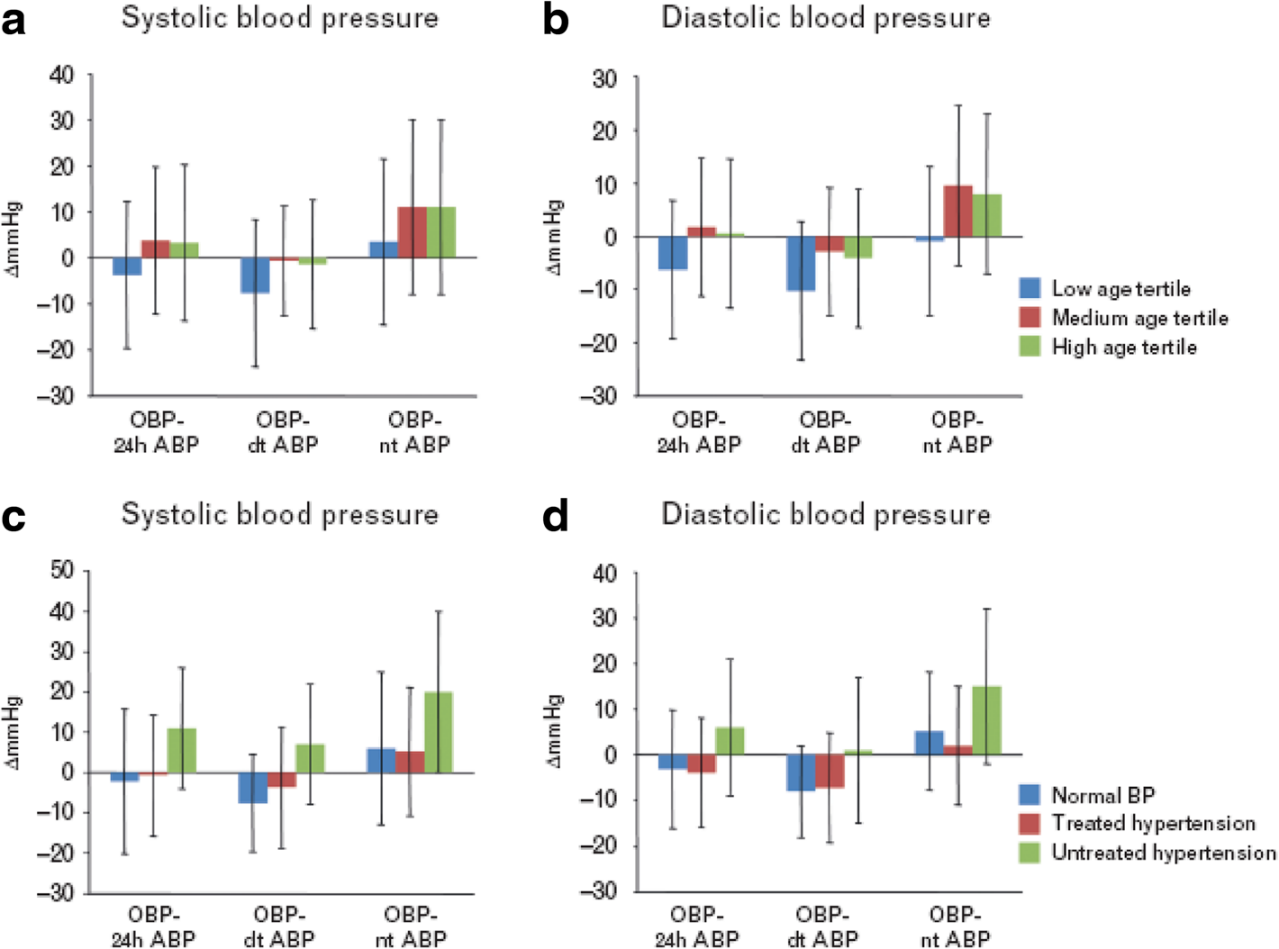
Differences between office and ambulatory blood pressure in children and adolescents Office blood pressure-ambulatory blood pressure differences in various subgroups of children (systolic values at left, diastolic values at right). **a** and **b** Groups defined by tertiles of age. Blue: low age tertile (4–10 years); red: medium age tertile (10–14 years); green: high-age tertile (14–18 years). **c** and **d** Groups defined by blood pressure status. Blue: normotensive patients (normal blood pressure in absence of treatment); red: treated hypertensive patients; green: untreated hypertensive patients. Numbers of patients in each subgroup are indicated in Table 1. The histograms represent means of the various types of differences listed at the bottom and bars represent _ SDs. Δ mmHg, differences in mmHg; 24-h ABP, 24-h mean of ambulatory blood pressure; dt-ABP, mean of daytime ambulatory blood pressure; nt-ABP, mean of night-time ambulatory blood pressure; OBP, office blood pressure. From Salice P. et al. [79] (permission required)

Children who present normal office, but elevated ABPM blood pressure values are defined as having masked hypertension. Some studies suggest that masked hypertension in children is associated with early organ damage and that it may be a precursor of sustained forms of hypertension [82, 83]. For this reason the American recommendations consider it appropriate to perform ABPM in subjects in whom this form of hypertension is suspected. Unfortunately the real prevalence of masked hypertension in children is not known and it would take many ABPM recordings in large non-selected populations to define it. The problem of the identification of children with white coat hypertension or masked hypertension is even more complicated by the recently published observations of Salice et al. [84]. In this important study it is shown how, in a high number of children, the correspondence between office and ABPM blood pressure values changes according to age and clinical conditions of the subjects (Fig. 2). While children in the low age range and normotensive children had higher systolic and diastolic blood pressure at ABPM than in the office, both older and hypertensive subjects showed lower ABPM values compared to the ones measured in the doctor’s office. These data suggest the need to pay particular attention in the diagnosis of white coat hypertension and masked hypertension.

##### Prehypertension

The term prehypertension used in this context should not be confused with the one that was used in the past to define those subjects presenting office blood pressure values above the 90^th^ but below the 95^th^ percentile and that was substituted in the most recent recommendations by the term “high-normal blood pressure”. In the present document prehypertension is defined (Table 5) as high-normal office blood pressure values, 24-h mean ABPM values below the 95^th^ percentile and a blood pressure load of 25 % of the measurements or higher. It should be noted that in the guidelines for adults the concept of diagnosis of prehypertension by ABPM does not exist, as in adults the assessment of pressure load is not used; in children however, this parameter is essential for the definition of prehypertension.

##### Diastolic hypertension

Another important new development in the 2014 recommendations is the introduction of diastolic blood pressure values for the diagnosis of hypertension. In the document of 2008 the diagnosis was based exclusively on systolic blood pressure. The diagnostic use of ABPM-derived diastolic blood pressure values can be important especially in children with secondary hypertension, in whom ABPM may reveal diastolic hypertension [85], whereas in children with primary hypertension ABPM more frequently indicates systolic hypertension [86, 87].

#### Technical problems regarding the correct use of Ambulatory Blood Pressure Monitoring in children

Nurse training is of fundamental importance for obtaining a competent and attentive staff, capable of correctly performing the technical part of the blood pressure measurements and using the available instruments in the appropriate manner. It is also essential that the nurse be instructed on how to establish a relationship with the children’s families in order to gain their full collaboration. The nurses should inform the parents on the way the examination will take place, teach them how to fill in the blood pressure diary, how to reassure the children and how to prepare them about the physical disturbance they might perceive during the blood pressure measurement. The choice of the cuff is also a crucial aspect. The operator should measure the circumference of the child’s arm on the central part and choose the appropriate cuff size. The width of the cuff should be ≥ 40 % of the arm circumference at a point midway between the acromion and the olecranon. Cuffs that are too small overestimate to an important degree, whereas large cuffs slightly underestimate blood pressure values; for this reason it is advisable to choose the larger cuff in case of doubt. The suggested frequency of the measurements is 3–4 per hour during daytime and 2–3 per hour during the night. At data analysis the first measurements after positioning of the cuff should be eliminated, anomalous data (outliers) should not be taken into consideration and all data lying outside the following intervals should be discarded (preferably directly by the reading software, or afterwards by the operator):

**Table:** 

Systolic blood pressure	< 60 or > 220 mm Hg
Diastolic blood pressure	< 35 or > 120 mm Hg
Heart rate	< 40 or > 180 beats per minute
Pulse pressure	< 40 or > 120 mm Hg

Once the 24-h systo-diastolic blood pressure tracing has been obtained, the data should be interpreted using the appropriate pediatric nomograms (Tables 6, 7, 8 and 9) and bearing in mind the technical aspects reported above.

## Conclusions

In pediatric age ABPM should not be used “routinely”. This test, can be extreme usefulness if used by skilled operators and if justified by specific questions (suspicion of white coat hypertension or masked hypertension, need for monitoring the effectiveness of a therapy, evaluation of the presence or absence of nocturnal dipping). The use of ABPM is particularly helpful in children aged more than 10 years. In younger children, who often do not tolerate the examination, ABPM should be used in targeted cases and bearing in mind that, in this age group, office blood pressure values are lower than those found with ABPM, and that this phenomenon tends to attenuate over the years. A future goal regarding ABPM in children will be to obtain more reference data for different age ranges and different ethnicities. It is also important to perform studies that assess the association between ambulatory blood pressure values and organ damage, not only by considering mean values, but also by examining anomalies regarding pressure load and the absence of physiological nocturnal dipping. Finally, it would be extremely useful to carry out prospective studies that could verify if normalization of 24-h blood pressure values is or is not accompanied by a regression of pre-existing organ damage.

### Hypertension and physical activity: about prevention, therapy and certifications

#### Introduction

Physical activity, be it playful and recreational or structured in organized sports, represents a fundamental cornerstone in the prevention and treatment of hypertension in pediatric age. This consideration, even though shared by all pediatricians and supported by broad evidence, is generally taken for granted and only briefly mentioned in the recommendations of the scientific societies dedicated to this subject. On the contrary, in the present update we dedicate a section to physical activity that is adequate and moreover proportional to our conviction that it is a primary and irreplaceable instrument in the prevention and treatment of hypertension in view of the well-known effects physical activity has on the pathogenetic mechanisms of hypertension. Furthermore, we will shortly deal with the legal aspects of the certifications for sports practice and the (few) contraindications for physical activity to be taken into consideration in children and adolescents with hypertension.

#### Prevention of hypertension and its risk factors by physical activity

The prevention of primary hypertension in children and adolescents is based on an intervention strategy on modifiable risk factors. Among these, the ones with the greatest impact are: overweight, diet, salt intake, sedentary behavior, bad sleep quality and cigarette smoking (also passive). Physical exercise may exert a possible effect on: overweight, sodium balance and bad quality of sleep, thus acting indirectly on the metabolic mechanisms involved in the development and maintenance of pressure increase, such as deposition and distribution of the fat mass, insulin resistance, activation of the sympathetic nervous system, sodium homeostasis, renin-angiotensin system, regulation of vascular function.

##### Obesity

Obesity is the main risk factor for hypertension in children and adolescents. A recent meta-analysis analyzed the effects of childhood obesity prevention programs on hypertension [88]. The most effective interventions, excluding the ones aiming at children who were already overweight or obese, were shown to be those combining dietary interventions with physical activity. The mean reduction in blood pressure obtained after 6–12 months with the combination of the two strategies was 1.64 mmHg for systolic and 1.44 mmHg for diastolic blood pressure. When intervening solely on diet or physical activity the results were shown to be less successful. For that matter, even if apparently quite modest, a reduction of blood pressure of 2 mmHg reduces the risk of hypertension in adulthood by up to 10 % [89]. Another meta-analysis examined nine randomized controlled trials in order to analyze the effect of physical exercise on blood pressure at rest in obese children [90]. The results revealed that programs comprising three exercise sessions a week, each lasting more than 60 min, induced a reduction in systolic blood pressure between −0.58 and −0.82 mmHg. Only if the frequency of exercise sessions was higher than three times a week it was possible to observe a reduction in diastolic blood pressure as well.

##### Sodium

On the average sodium intake in children and adolescents exceeds the recommended quantities [91]. Physical activity practiced on a regular basis induces sodium loss through sweating, thus favoring the achievement and/or maintenance the equilibrium of the sodium balance [92]. If the level of exercise is intensified, sweating and consequently sodium loss increases. The recommendation for practice of physical activity in children at the Centers for Disease Control and Prevention USA indicates at least 60 min daily of moderate or intense activity, with at least three sessions of vigorous activity a week [93]. The many different personal and environmental factors influencing the amount of sweat produced make it difficult to quantify the relative impact of sodium loss by sweating due to physical activity on the overall effect of physical exercise on blood pressure.

##### Sleep

Duration of sleep is inversely correlated to cardio-metabolic risk, obesity and levels of physical activity in children [94, 95]. Children that sleep more than 9 h a night present a more intense physical activity level and are leaner than children that sleep less. However, it is not known if it is the higher level of motor activity (duration and/or intensity) that causes an increase in duration of sleep or *vice versa* [90]. Also the regularity of sleep duration during weekdays and the weekend is related to a higher level of physical activity [96]. The amount of time spent watching TV or playing videogames is found to be associated with a shorter duration of sleep [97].

#### Physical activity in the treatment of children and adolescents with hypertension

The benefits of physical activity for the treatment of hypertension have been well documented for what regards hypertensive adults, both with and without obesity [98, 99]. On the contrary evidence is less strong in pediatric age, in which the majority of meta-analyses and systematic reviews focus on the effectiveness of physical activity on blood pressure values in obese children and adolescents, as obesity represents the most frequent cause of hypertension in this age range. The mechanisms by which physical activity may affect blood pressure in obese children and adolescents are not yet well known. It is hypothesized that physical exercise, i.e., physical activity performed in a structured manner according to precise criteria regarding type, duration, intensity and frequency, acts on various mechanisms involved in the onset of hypertension, determining a reduction in insulin resistance and beneficial adaptation of the cardiovascular system (reduction in sympathetic tone, reduction in arterial stiffness, reduction in endothelial dysfunction) [100]. Furthermore, reduction in body mass index (BMI), due to the negative caloric balance achieved when physical activity is associated with a diet, can cause a reduction in blood pressure of 8–12 mmHg [101]. Some observational studies demonstrated a significant relationship between active lifestyle, aerobic fitness and levels of blood pressure [102]. Therefore, lifestyle changes by increasing physical activity and reducing sedentary behavior represent the first intervention strategy in pediatric hypertension (in combination with diet, reduction of stress factors, and avoidance of smoke and alcohol) in patients with high-normal blood pressure or with stage 1 hypertension [103]; at the same time, such interventions represent a complementary and irreplaceable therapy in those cases in which pharmacological treatment is needed. In order to achieve the highest probability of success, the whole family should participate in changing the dysfunctional behavior. For what concerns the type, intensity and duration of physical exercise to be advised in the treatment of pediatric hypertension, experimental studies have shown a greater efficacy of aerobic exercise (systolic blood pressure −1.39 mmHg; diastolic blood pressure −0.39 mmHg) compared to muscular strength training (systolic blood pressure −0.61 mmHg; diastolic blood pressure −0.51 mmHg) [90]. The training programs based on three weekly sessions lasting more than 60 min each, were found to be more effective on systolic blood pressure, while programs with more than three sessions per week had a greater impact on diastolic blood pressure. Even though the effect on blood pressure values may seem quite modest, it has been hypothesized that the reduction of at little as 1 mmHg in childhood may have an impact on the blood pressure in adult age and on future cardiovascular events, in view of the long cumulative period of exposure [104]. A meta-analysis of studies in adults has shown that a reduction of blood pressure by 2 mmHg can reduce cardiovascular event risk by 12 % [105]. Therefore, on the basis of scientific evidence, it is recommended to practice physical activity for at least 5 days a week, lasting 30–60 min at moderate to vigorous intensity [106] (moderate physical activity: reaching 55–75 % of maximum heart rate; vigorous physical activity: reaching 65–85 % of maximum heart rate). The physical activity can be performed both as a non-organized activity and as a physical exercise or sport. In case a condition of elevated blood pressure is diagnosed in children that already participate in one or more physical activities, it may be necessary to increase the frequency and/or intensity. Aerobic activities are to be preferred (walking, running, cycling, swimming, rowing) over static forms of exercise. Moreover, considering the negative and independent effect of sedentary behavior on cardio-metabolic health, children and adolescents should be educated to monitor the time spent in sedentary activities (TV, videogames, computer), aiming at gradually reducing the time dedicated to these activities to less than 2 h a day [107]. Significant effects of exercise have been shown after 3–6 months from the start of the training program [108], but this should continue in time in order to obtain a more lasting effect on blood pressure control. It is also recommended not to interrupt the physical exercise programs, as blood pressure returns to pre-training values within a few months after the cessation of the exercise.

#### Aspects related to the certification for physical activities and contraindications for sports activities in hypertensive children and adolescents

**Fig. 3:**
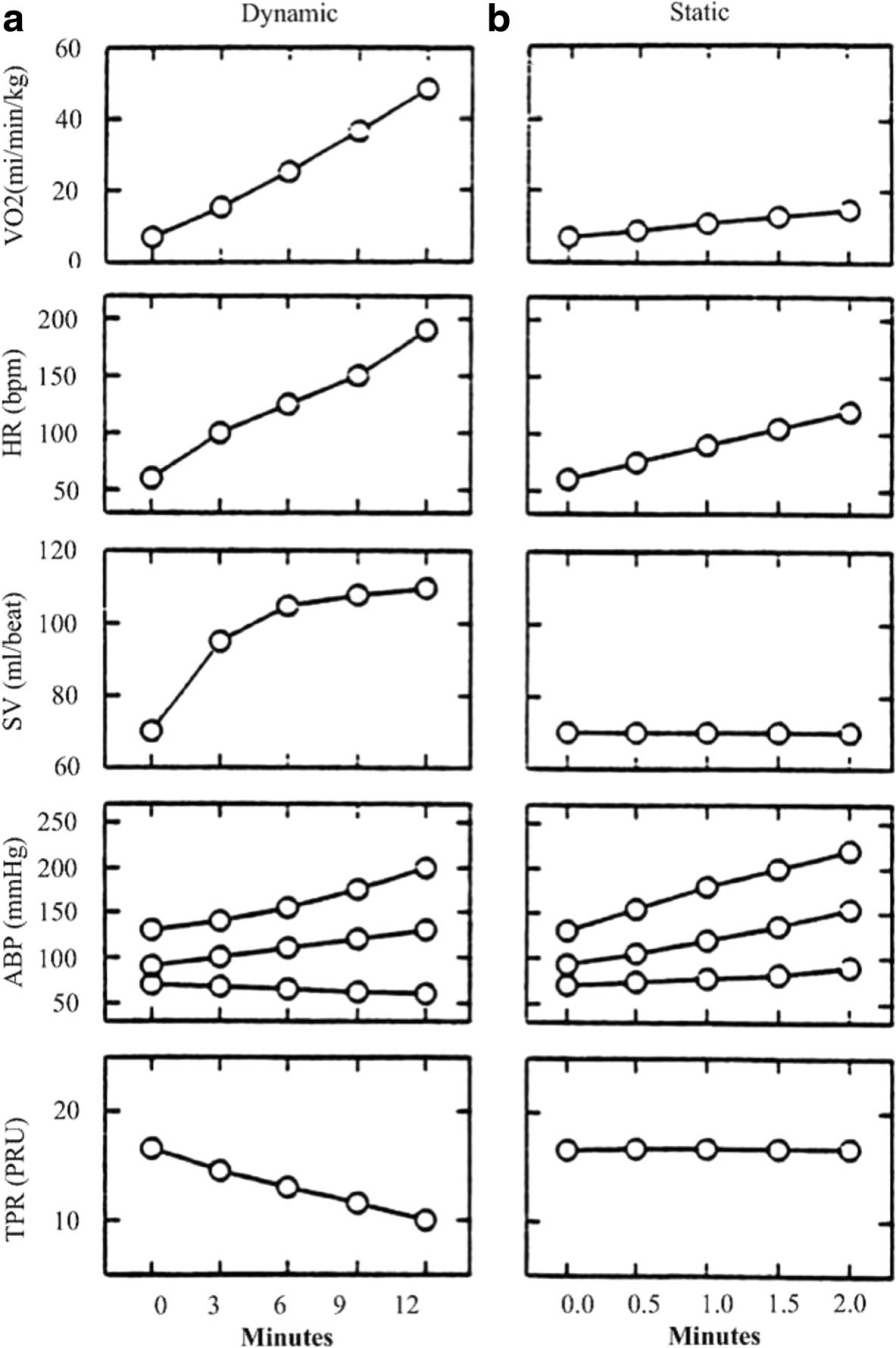
Cardiovascular response to physical exercise according to type of exercise. **a** Response to dynamic exercise with progressively increasing workload up to maximal oxygen consumption. **b** Response to dynamic exercise (handgrip at 30 % of the maximal voluntary contraction). VO2 (ml/min/kg): oxygen consumption; HR (bpm): Heart Rate; SV (ml/beat): Stroke Volume; ABP (mmHg): systolic, diastolic and mean blood pressure; TPR (PRU): Total Peripheral Resistance (expressed in Peripheral Resistance Unit) http://content.onlinejacc.org/data/Journals/JAC/23038/02015.pdf

Several scientific studies confirm that the level of physical activity is inversely proportional to mortality from cardiovascular causes both in men and women. This consideration holds true for hypertensive subjects as well, in whom the absence of physical activity increase the risk of cardiovascular diseases. It is well known that blood pressure control during physical activity is a complex process, involving increase in stroke volume and heart rate, changes in the peripheral vascular resistances and altered sympathetic tone. All these adaptations, always depending on one another, are related both to the type of physical exercise and to its intensity and duration. They are also affected by gender, with more marked changes in males compared to females, but also by the proportion between lean mass and fat mass. Furthermore it is necessary to distinguish physical exercise in two main types: dynamic and isometric (or static). The first type of exercise causes an increase in cardiac output associated with rapid increases in heart rate and systolic blood pressure (with slight decrease in diastolic blood pressure), but also with significant reductions in peripheral vascular resistance. Recordings have been reported hat show systolic blood pressure values up to 250 mmHg during treadmill exercise stress test in young healthy males [109]. The second type, isometric or static exercise induces a sudden increase in both systolic and diastolic blood pressure, a modest increase in heart rate with an unchanged or slightly reduced stroke volume, but no reduction in peripheral vascular resistance. In young male weightlifters extremely high values of systolic blood pressure up to 300–400 mmHg have been recorded by direct intra-arterial measurements [110]. Figure 3 shows the changes and interactions between the different parameters during static and dynamic exercise [111]. From this preamble it emerges that it is necessary that young hypertensives are stimulated to practice the kind of sports activity that is appropriate for their individual cardiovascular condition. In 2010 the American Academy of Pediatrics Council on Sports Medicine and Fitness released new recommendations for the participation in sports activities by children and adolescents who have systemic hypertension [112]. In case of high-normal blood pressure lifestyle changes should always be encouraged (reduction of overweight and of dietary sodium intake), avoiding the use/abuse of illegal substances or at least those inducing an increase in blood pressure (hormones, ephedrine, tobacco or alcohol) and limiting the consumption of energy or caffeine containing drinks. At this blood pressure level there are no particular restrictions to the type of sports activity, but a check of blood pressure values, at least twice a year, should be recommended. In case of stage 1 hypertension, besides following the recommendations that count for high-normal blood pressure, an accurate assessment should be added aiming at excluding the presence of organ damage and, in selected cases, medical treatment may be initiated that may not have any negative effect on the performance (beta-blockers and diuretics should be avoided; diuretics are considered “doping”). In stage 2 hypertension, even in the absence of organ damage, it is not recommended to practice sports that are mainly isometric and at high intensity, such as weight lifting and push-ups, as they cause sudden steep increases in blood pressure. As holds for stage 1 hypertension, also in this case, pharmacological treatment should be taken into consideration in an adequate manner. Children with high-normal blood pressure, with stage 1 hypertension without organ damage or with pharmacologically controlled stage 2 hypertension can participate in sports at the agonistic level. The explanatory Table 10 (extracted from a document of the “Comitato Organizzativo Cardiologico per l’Idoneità allo Sport” [113]) reports a list of sporting activities characterized by mainly pressure-related cardiocirculatory involvement. This does not mean that hypertensive children cannot occasionally practice these disciplines in a recreational way. However, it is advisable that the pediatrician knows that these sports are not suitable for subjects with hypertension, not only in view of the certification for practicing non-agonistic sports, but also to avoid that children are introduced in a certain discipline that afterwards they will not be able to perform in an agonistic manner.

**Table 10:** Sporting activities with mainly pressure-related cardiocirculatory involvement, characterized by increased-to-maximal heart rate, mean-to-increased peripheral resistances and a non-maximal cardiac output

Mountaineering, Climbing
Athletics: speed, jumps, leaps, heptathlon, decathlon
Bobsledding, Luge
Weight lifting, Bodybuidling
Speed cycling, Mountain bike downhill, BMX
Artistic gymnastics
Synchronized swimming
Motorcycling (Motocross, Enduro, Trial)
Water skiing
Skiing: slalom, giant slalom, super G, downhill, alpine, snowboard, carving, grass skiing.
Surfing
Tug-of-war

N.B. The following activities can be carried out occasionally and in a recreative manner also by hypertensive children or adolescents. However, it is advisable that the Pediatrician does not stimulate them to practice these sports permanently; moreover, the children would not obtain the medical certificate required for these activities to be performed as competitive sports

Comitato Organizzativo Cardiologico per l’Idoneità allo Sport ANCE, ANMCO, FMSI, SIC, SIC SPORT [113]

In Italy, unlike what happens in the United States, in order to engage in any non-agonistic sports activity a medical certification is requested, that involves civil and penal responsibilities of the certifying doctor and that is regulated by specific decrees, in particular by the most recent changes described in the so-called Balduzzi decree [114]. For obtaining this certification in healthy, or apparently healthy children, as defined in generic terms, blood pressure measurement and at least one electrocardiogram at rest should be performed (as explicitly specified in the wording on the certificate to be signed afterwards by the physician). In the case of children and adolescents suffering from hypertension, both primary and secondary, it is necessary to pay more attention, both considering the cardiovascular stress that this state may involve, and considering the importance of implementing the lifestyle changes, that should always represent the first, non-pharmacological, step that may be useful in maintaining blood pressure values within the normal range. In special cases (e.g., obese patient losing weight) it is possible to certify suitability for moderate non-agonistic sports, with a frequency of maximum 2–3 h a week in order to attain improvement in lifestyle without at the same time weighing negatively on the cardiovascular and osteoarticular apparatus. On the other hand, for participation in agonistic sports activities, children have to be referred to Sports Medicine Specialists, who are qualified to decide what examinations should be performed in order to obtain certification of suitability for the specific sport and who finally sign the certificate.

### Simple carbohydrates, fructose, uric acid and hypertension

In this section we will deal with the relationship between blood pressure, simple carbohydrates (fructose in particular) and uric acid in pediatric age. As the metabolisms of these compounds are strongly interconnected, it is not easy to distinguish the specific role of each of them. Furthermore, they are also associated with parameters that characterize the metabolic syndrome, such as hypertriglyceridemia, low levels of HDL-cholesterol, hyperinsulinemia, type 2 diabetes mellitus, fatty liver, in addition to their role in the development and progression of renal disease.

For the sake of clarity each compound is taken into consideration separately.

#### Simple carbohydrates

This term comprehends both the monosaccharides glucose and fructose and the disaccharide sucrose, all used in the food industry, in homemade preparations or directly by the consumer and which are also the sugars naturally contained in honey, syrups, juices or fruit concentrates. One molecule of glucose bound to one of fructose by a glycosidic linkage form one molecule of sucrose, the common table sugar. In 2000 the dietary consumption of simple carbohydrates in the Unites States was found to amount to 72.6 kg/person/year and it has been calculated that about 40 % of this total was accounted for by sweetened beverages [115]. This consumption, involving children to a large extent, introduces a considerable caloric amount and may be a decisive contributing factor to the current epidemics of overweight [116], considering the fact that calories originating from liquids do not tend to cause a sense of satiety, whereas those in solid foods normally do [117, 118]. In these cases we are talking about an additional caloric intake up to about 200–300 Kcal a day. The relationship between overweight and hypertension in pediatric age is well-known and has been widely discussed in our previous article [119]. Furthermore it has been shown that sugar consumption is positively correlated with salt intake, both in adults and in children [120, 121], and this could represent an additional mechanism favoring elevated blood pressure values.

#### Fructose

Fructose is the sugar that can be found in fruit, but in the present-day diet, the amount of fructose actually deriving from fruit is quite modest. An important proportion of the intake of fructose derives from sucrose, while another major source of fructose is represented by high fructose corn syrup (HFCS). This is an industrially produced syrup derived from corn starch, introduced in the seventies, which contains 55-60 % of fructose and is obtained by isomerization of glucose. These syrups are frequently used in the food industry for the production of various prepackaged foods that do not necessarily have a sweet taste, like crackers or ketchup, but are actually mainly employed in the production of sweetened beverages. Finally, currently fructose is also being used in several sweeteners. A moderate fructose intake, especially when deriving from fruit consumption, is well tolerated. What does cause quite a lot of concern, even though not yet shared by the public opinion, is an excessive fructose load as the human body does not envisage any system to regulate its metabolism. Fructose can be internalized by all types of cells, but the mechanisms of intracellular transport of this carbohydrate are most efficient in the liver and kidneys, and so these two organs are mainly involved in its metabolism. Unlike what happens with glucose, the intracellular use of fructose does not depend on any control system that considers the energetic state of the cell. As a consequence, abundant amounts of fructose induce an uncontrolled cascade synthesis of fatty acids, whereas equivalent amounts of glucose would not cause such effects [122]. Furthermore, when entering the cell fructose is phosphorylated by an ATP-dependent fructokinase. The presence of an excess amount of fructose causes an increased ATP consumption, that cannot be restored due to a relative phosphorus deficiency and in turn this leads to progressive degradation of the high-energy compounds to metabolized adenosine and finally uric acid [123]. Moreover, it is suggested that large amounts of fructose induce an increased activity of the enzyme fructokinase, while fructose itself may also reduce renal secretion of urates [124]. The final result of these interlinked effects is an increase in blood uric acid after excessive consumption of fructose. The consequences of high uric acid levels will be analyzed separately. Compared to glucose, fructose appears to cause a weaker sense of satiety due to its inability to increase insulin and leptin secretion and to inhibit ghrelin secretion, which are all compounds having a stimulating or inhibiting effect on the satiety center in the brain [125]. Fructose is also believed to decrease basal metabolism [126]. It increases reabsorption of salt and water in the intestine and in the kidneys and it has been shown that a diet at high contents of both fructose and sodium together is more easily associated with the development of hypertension than a solely high-fructose diet [127]. Fructose has also been reported to induce oxidative stress and to provoke vasoconstriction by inhibiting endothelial nitric oxide synthase [128]. In experimental animals also a negative effect of fructose on the kidneys has been revealed, inducing the development of renal hypertrophy, glomerular hypertension, cortical vasoconstriction and preglomerular arteriolopathy. If high fructose intake is maintained for a long time, the renal damage, which may be modest in the initial phases, will tend to become chronic and may cause systemic hypertension in the long run [129]. Although a marked increase in the risk of gout has been demonstrated in subjects with a particularly high-fructose diet, this event is very rare in young individuals. In spite of this, the intake of sugar-sweetened drinks is associated with an increased risk of hyperuricemia [130] and these observations have been reconfirmed in a vast cohort of children and adolescents [131], in whom an increased intake of these kind of beverages corresponded with a significant increase both in uric acid levels and in blood pressure even after correction for potential confounding factors such as BMI. These data suggest other mechanisms, besides through obesity, by which sugar-sweetened drinks may play a role in the development of hypertension in the adolescents. Intervention studies have shown that reduction of carbohydrate consumption can slow the development of the metabolic syndrome more effectively than the typical diets that mainly reduce intake of fat. A study in California has demonstrated that banning soft drinks in schools caused a reduction in their daily consumption associated with a significant reduction of obesity in children between 6 and 11 years of age [132].

#### Uric acid

Uric acid is produced during the breakdown of the purine bases adenine and guanine. A diet containing food at high concentrations of these compounds can bring about an increase in uric acid level in the blood, especially in the presence of minor metabolic dysfunctions in specific subjects. However, one should not forget the role that excessive fructose intake can have in increasing uric acid (see previous paragraph). Uric acid can exert negative effects even at concentrations that are lower than the ones provoking precipitation of urate crystals in the joints. In children and adolescents it is advised to attentively examine also uric acid levels that are at the upper limit of the normal range. The relationship between uric acid levels and blood pressure values was hypothesized many years ago, but lately research in this field has become quite strong, producing numerous data deriving from both animal and human studies, some with interesting results in pediatric age [133]. In experimental moderately hyperuricemic rats it has been observed that uric acid reduces the production of nitric oxide both at endothelial and renal level with a consequent vasoconstriction in these particular areas [134]. At vascular level uric acid stimulates smooth muscle cell proliferation, whereas it inhibits production of new endothelial cells. These effects, typically involved in what is generally called endothelial dysfunction, seem to cause blood pressure elevations and reductions in blood flow to the skeletal muscle, that, consequently, needs more insulin for the uptake and disposal of excess glucose, resulting in a clear-cut effect of insulin resistance. Furthermore, such vascular alterations induce arteriolar hyalinosis, described mainly at the level of the afferent arterioles of mildly hyperuricemic rats, in which a further activation of the renin-angiotensin-aldosterone system and the onset of sodium sensitivity have been observed, both factors that clearly contribute to the development of hypertension. The comprehensive data deriving from experimental studies performed in both animals and humans suggest the existence of two steps in the pathogenetic mechanisms that link uric acid to the development of hypertension [135]. Initially uric acid activates the renin-angiotensin-aldosterone system and suppresses nitric oxide, causing a functional increase in the vascular resistances at systemic level. This is followed by a second phase characterized by the development of arteriosclerosis of the afferent glomerular arterioles and the onset of sodium sensitivity, which are both structural changes and thus hardly reversible. Uric acid presents antioxidant properties in the extracellular space, suggesting that it may play a protective role against some disease conditions. Nevertheless, many studies have shown that once uric acid enters the cells, it starts to assume a pro-inflammatory role. Intracellular uptake of uric acid is mediated by the transporter URAT1. At smooth muscle cell level, the entry of uric acid causes activation and production of growth factors and of MCP-1 [136], which is a chemokine involved in the formation of inflammatory infiltrates and implied in the development of insulin resistance, because of its additional capacity to reduce glucose uptake at the myocyte level. Internalization of uric acid in adipocytes through URAT1 has been found on the one hand to induce MCP-1 production and on the other hand to reduce production of insulin sensitizers such as PPARγ and adiponectin [137], thus contributing in an even more structured manner to the development of insulin resistance. In the kidneys, hyperuricemia has particularly clear effects in animals with pre-existing renal disease as it can aggravate both glomerulosclerosis and tubulointerstitial damage [138]. Instead, lowering uric acid by drug treatment has been demonstrated to attenuate the progression of renal disease and hypertension [139]. Entry of uric acid in human proximal renal tubule cells causes apoptosis due to a mechanism that is mediated by the activation of pro-oxidant systems [140] and this may explain, at least partly, the results of clinical studies describing how even moderately increased uric acid levels are associated both with the onset and with the progression of chronic renal disease in different clinical settings [141]. Non-alcoholic fatty liver disease (NAFLD) is the result of abnormal fat accumulation in the liver and has recently been found to be strongly related to the presence of insulin resistance, metabolic syndrome and increases uric acid levels. Indeed, uric acid contributes both to lipoprotein oxidation and to inflammation, two important factors in the development and progression of NAFLD. The association between NAFLD and unfavorable cardiovascular risk profile is increasingly supported by scientific evidence also in the pediatric population [142]. Preliminary data in animal models suggest that hypouricemic therapy may improve the severity of steatosis [143]. In humans the increase in uric acid has been demonstrated to be an independent predictor of obesity, hyperinsulinemia, diabetes, systemic hypertension, cardiovascular and renal diseases [144]. In particular, a recent meta-analysis shows that the relationship between uric acid levels and the development of hypertension is dose-dependent [145]. Some recent publications also concerned subjects in the pediatric age range. Various authors described the contemporaneous presence of high uric acid levels and metabolic alterations [146]. Ford reported that an association between uric acid and components of the metabolic syndrome, particular visceral obesity, is already present in children and adolescents. In obese children also a significant correlation between uric acid plasma levels, blood pressure and HOMA index has been reported, suggesting a possible role of uric acid in the pathogenesis of pediatric essential hypertension mediated by the presence of insulin resistance [147]. A close relationship between uric acid levels and components of the metabolic syndrome has also been shown in a population of Japanese adolescents [148]. Feig was the first author to describe an association between uricemia and the presence of hypertension in a cohort of children and adolescents [149]. It is interesting to emphasize that elevated urate levels were found in subjects with primary hypertension. Jones [150] confirmed the relationship between uric acid and blood pressure levels in a cohort of 104 children with primary hypertension, undergoing 24 h ambulatory blood pressure monitoring. This relationship persisted even after correction for age, gender, race and BMI. Afterwards other authors strengthened the evidence of this type of association. Particularly worthy of note is a study derived from the US registers (NHANES), demonstrating in over 6000 adolescents that those who had hypertension showed higher uric acid levels [151]. Recently, in a cohort of children at relatively high cardiovascular risk, a significant correlation has been described between increasing levels of uric acid and the grade/category of essential hypertension (transient hypertension, prehypertension and sustained hypertension), which persisted after correction for possible confounders, including HOMA-index [152]. It has also been suggested that minor increases in uricemia in children may predict the onset of hypertension in adulthood [153]. At this moment the role of uric acid as risk factor for the development of cardiovascular and renal damage in very young children is being studied. There are some preliminary reports indicating a higher prevalence of carotid atherosclerosis in adolescents with increased uric acid levels [142]. The relationship between uric acid and renal tubular damage has recently been demonstrated also in a population of hypertensive adolescents with normal renal function, in whom a slight increase in uric acid correlated with an increased urinary excretion of early markers of renal injury such as NGAL and KIM-1 [154]. Two randomized studies in hypertensive and prehypertensive adolescents have revealed a reduction of systolic and diastolic blood pressure values coinciding with the administration of uric acid lowering drugs, suggesting a direct causal relationship between urate and hypertension in these subjects [155, 156]. Further studies are needed in order to understand if uric acid lowering pharmacological treatment should be taken into consideration for hypertensive children. Recent findings support the view that maintaining relatively low uric acid values over time could be more effective at preventing the onset of hypertension than lowering uric acid, to reduce blood pressure levels once hypertension is established [157]. However, further studies are needed in order to understand if uric acid is really implicated in the pathogenesis of essential hypertension in children and adolescents.

From the foregoing the consequences in terms of both prevention and dietary treatment of hypertension in children are quite clear. The recommended quantities of food intake for the Italian population (http://www.sinu.it/html/pag/tabelle_larn_2014_rev.asp) affirm that the consumption of simple carbohydrates should not exceed 15 % of the total caloric intake, whereas the World Health Organization (WHO) limits this quote to only 10 % and suggests that 5 % would be ideally preferred (http://www.who.int/mediacentre/news/releases/2015/sugar-guideline/en). A recent study in Italian children of 0–36 months of age showed that as early as at six months of age only one third of the children presented a carbohydrate intake that was lower than 15 % of the total calories, while from 9 months on almost all children had an intake that exceeded this value [158]. Food with a high glycemic index, such as homogenized baby food or fruit juice with added simple carbohydrates, contribute largely to this situation. Fortunately some baby food companies are becoming aware of the problem and have started to promote products without added simple sugars. In any case it would be advisable to use fresh fruit instead of prepackaged products already from weaning onwards. Even if in Italy the consumption per person of simple carbohydrates seems to be lower than that observed in the United States of America, in order to prevent obesity, hypertension and metabolic syndrome, it would be necessary to take measures to restrain the wide consumption of these foods, and beverages in particular. Specifically, the use of fructose, that is found in HFCS and employed as a sweetener in soft drinks, juices or fruit drinks, should be limited. In hypertensive and prehypertensive children an accurate nutritional assessment of the consumption of simple sugars should be carried out. The intake of these compounds should be reduced to the amount recommended by the LARN and it would be advisable to eliminate sweetened beverages completely. In subjects at risk of hypertension it would also be appropriate to measure uric acid concentrations and to carefully examine also those values that are in the high-normal range. In those cases it would be better to avoid high-purine foods (Table 11) as no pharmacological treatment can be proposed, besides reducing the intake of fructose even more attentively.

**Table 11:** Foods to pay attention to in case of elevated serum levels of uric acid

Foods at very high purine content (avoid)
Anchovies, brains, game, gravy, herring, kidney, liver, sardines, shellfish, sweetbread.
Foods at high purine content (limit)
Asparagus, beef stock, cauliflower, eel, legumes (beans, lentils, peas), meat (beef, lamb, pork, veal), mushrooms, oatmeal, wheat bran, wheat germ.
